# Incidence and risk factors of retinopathy of prematurity in an Italian cohort of preterm infants

**DOI:** 10.1186/s13052-021-01011-w

**Published:** 2021-03-12

**Authors:** Carlo Dani, Caterina Coviello, Fiorenza Panin, Saverio Frosini, Simonetta Costa, Velia Purcaro, Domenico Lepore, Giovanni Vento

**Affiliations:** 1 Division of Neonatology, Careggi University Hospital, Largo Brambilla 3, 50141 Florence, Italy; 2 Department of Neurosciences, Psychology, Drug Research and Child Health, Careggi University Hospital of Florence, Florence, Italy; 3 Eye Clinic, Neuromuscular and Sense Organs Department, Careggi University Hospital, Florence, Italy; 4 Division of Neonatology, Department of Woman and Child Health and Public Health, Fondazione Policlinico Universitario A. Gemelli, IRCCS, Catholic University of Sacred Heart, Rome, Italy; 5 Department of Ophthalmology, Gemelli Foundation IRCSS, Catholic University of the Sacred Heart, Rome, Italy

**Keywords:** Retinopathy of prematurity, Risk factors, Human milk, Interventricular hemorrhage, Preterm infants

## Abstract

**Objective:**

Non-negligible differences in retinopathy of prematurity (ROP) and its risk factors between different neonatal intensive care units (NICUs) are reported. Our aim was to assess the incidence and risk factors for ROP development in a large cohort of very preterm infants who were assisted in two Italian NICUs.

**Methods:**

Preterm infants with gestational age between 23^+ 0^ and 29^+ 6^ weeks were stratified into subgroups of infants who developed ROP and those who did not; their clinical characteristics were compared with univariate and multivariable logistic regression analyses.

**Results:**

We studied a total of 178 infants of whom 67 (38%) developed ROP (stage 1: *n* = 12; stage 2: *n* = 41; stage 3: *n* = 14). Regression analysis demonstrated that maternal milk (OR 0.979, 95% Cl 0.961–0.998) decreased the risk of developing ROP, while intraventricular hemorrhage (IVH) (OR 2.055, 95% Cl 1.120–3.772) increased it. Moreover, maternal milk was found to decrease (OR 0.981, 95% Cl 0.964–0.997) the risk of ROP at discharge, while RBC transfusion increased it (OR 1.522, 95% Cl 1.208–1.916).

**Conclusions:**

In our cohort the occurrence of ROP was similar to that previously reported. Strategies for promoting the use of mother’s own milk, preventing IVH, and standardizing the approach to RBC transfusions could contribute to decreasing the risk of ROP in very preterm infants.

## Background

Retinopathy of prematurity (ROP) is a vasoproliferative retinal disorder, which is the main cause of visual impairment and blindness in preterm infants [1]. It has been reported that some stages of ROP occur in 40–50% of infants born < 30 weeks of gestational age, while severe ROP occurs in 7–8%, and treatment is needed in 5–6% [2].

ROP is a two phase disease with a first phase of reduced retinal vascular growth and loss of blood vessels due to a lack of growth factors and abnormal oxygenation [3, 4]. During this phase, the retina is incompletely vascularized with the development of a peripheral avascular zone which becomes increasingly hypoxic. This hypoxia induces the second phase of ROP characterized by an increase in the expression of the vascular endothelial growth factor (VEGF) which can promote uncontrolled neovascularization and retinal detachment [3, 4].

The most common treatment of severe ROP is retinal ablation using laser photocoagulation to reduce VEGF production of the hypoxic peripheral retina, but this treatment destroys approximately two-thirds of the retina [3, 5]. Over the last few years, intravitreal injections of anti-VEGF have emerged as an effective first-line treatment for severe ROP, and many authors have reported favourable outcomes using these drugs [2, 3, 6].

Recently, many studies have investigated risk factors and co-morbidities associated to ROP [7–14], but it is worth noting that large differences in ROP frequency are reported between different neonatal intensive care units (NICUs). Moreover, it is important to observe that risk factors have a significant etiopathogenetic effect in some units or countries while in other settings they do not. This suggests that changes in neonatal assistance could contribute to the decreasing the occurrence of ROP and that evaluation of local risk factors is necessary for correction and possible prevention of this severe complication.

Thus, the purpose of this study was to assess the incidence of ROP and to investigate risk factors for its development in a large cohort of very preterm infants who were assisted in two Italian neonatal intensive care units (NICUs). Our aim was also to compare these risk factors to those individuated in previous studies and, possibly, to individuate proper strategies to limit their effects.

## Methods

### Study population

This retrospective study was carried out at the third level NICUs of Careggi University Hospital of Florence and Fondazione Policlinico Universitario A. Gemelli of Rome, after the approval of local ethics committees. These NICUs assist about 3300 and 4000 newborns per year and have 16 and 10 beds for neonatal intensive care, respectively. Moreover, in these NICUs about 50 and 40 preterm infants are born with gestational age < 30 weeks per year, respectively, and there are about 0.5 cases of perinatal asphyxia per 1000 live births per year.

Infants were included in the study if they were born between 23^+ 0^ and 29^+ 6^ weeks of gestation. Exclusion criteria were major congenital malformations, chromosomal disorders, inherited metabolic diseases, and death before ROP screening.

### Study design

Serial eye examinations were performed by board-certified consultant ophthalmologists; the first examination was made according to the recommendation of the American Academy of Pediatrics on ROP screening [15]. ROP staging was performed in agreement with international classification [16]. When examination was normal, subsequent controls were scheduled every two weeks until complete retinal vascularization; if the vascularization was limited to zone 1 or in case of stage 1 and 2 the next ROP controls were weekly; in case of more severe forms of ROP (stage > 3 or plus diseases) eye examinations were performed several times a week at the discretion of the ophthalmologist. Eye examinations were performed through indirect ophthalmoscopy. The RetCam Imaging System II (Clarity Medical Systems, Pleasanton, CA) was used to confirm diagnosis, monitor disease and treatment, evaluate retinal neo-vascularization, and to take fundus photography in any grade ROP.

Clinical and demographic data were collected by reviewing patients’ medical records. For each studied infant, we reported the diagnosis of ROP, age at diagnosis, its stage, possible treatment, and outcome. Moreover, the following data were recorded: gestational age, birth weight, birth weight < 10° percentile, birth weight Z score, sex, Apgar score at 5 min, mode of delivery, need and duration of non-invasive respiratory support [high-flow nasal cannulae (HFNC), nasal continuous airway pressure (NCPAP), bi-level NCPAP (BiPAP), nasal intermittent mechanical ventilation (N-IMV)], and mechanical ventilation [patient-triggered ventilation or high frequency ventilation (HFV)]; number of red blood cell (RBC) transfusions, treatment with sildenafil, weight percentile at 36 weeks of post-menstrual age, percentage of human, maternal, and formula milk administered during hospital stay, occurrence of PDA requiring treatment, sepsis, bronchopulmonary dysplasia (BPD), necrotizing enterocolitis (NEC), intraventricular hemorrhage (IVH), periventricular leukomalacia (PVL), duration of hospital stay, and death. To evaluate birth percentile INeS growth charts were used [17]; RBC transfusions were decided according to the guidelines of the Italian Society of Neonatology [18]; BPD was defined as oxygen requirement at 36 weeks of post-menstrual age [19]; PDA was diagnosed by echocardiography; NEC was diagnosed according to Bell’s criteria [20]; sepsis was defined as positive blood culture; IVH was classified according to the Papile’s classification scheme [21]; and PVL was diagnosed according to the De Vries’ criteria [22].

All infants followed the same enteral nutrition protocol: trophic feeding was initiated within 24 h after birth and was continued at 20–40 mL/kg/d as tolerated for up to five days. Subsequently, the amount was increased by 20 ml/kg every day if enteral nutrition was tolerated. The goals for enteral nutrition were 150 mL/kg/d and 120 kcal/kg/d. Preterm formula was administered only when mother’s or donor’s human milk were not available.

The objective of respiratory support was to maintain PaO_2_ from 50 to 70 mmHg, PaCO_2_ less than 65 mmHg, pH > 7.20, and SpO_2_ from 90 to 95%. Surfactant was given as early rescue treatment.

### Statistical analysis

The infants enrolled in the study were stratified into subgroups of infants who developed ROP or those who did not. Their clinical characteristics were described as mean and standard deviation, median and range, or rate and percentage. Normality of data distribution was assessed by Shapiro-Wilk’s test. Parametric continuous variables were analyzed by the Student’s “t” test or by Wilcoxon rank sum test in case of deviation from normality assumptions. Categorical variables were compared using the Χ^2^ test. *P* < 0.05 was considered statistically significant.

A multivariable stepwise logistic regression analysis was performed to evaluate the potential independent effect of more important variables on the occurrence of ROP. We selected variables that at univariate analyses were different between the groups (*P* < 0.200) excluding those which were found to be collinear by calculating variance inflation factors (VIF). Thus, we assessed the effects of gestational age, birth weight (z-score), BPD, PDA, sepsis, NEC, IVH, PVL, RBC transfusions, maternal milk feeding, and treatment with sildenafil. A further multivariable stepwise logistic regression analysis was performed to evaluate the potential independent effect of the same variables on the ROP stage at discharge. Effect estimates were expressed as odds ratio (OR) with profile likelihood-based 95% confidence limits.

Data analysis was performed using IBM SPSS Statistics version 20 (SPSS INC, Chicago, Illinois, USA).

## Results

The study ran from November 2017 to March 2020. We studied a total of 178 infants of which 67 (38%) developed any grade of ROP. Diagnosis of ROP was made at the mean post-menstrual age of 33.4 + 1.7 weeks. Twelve (18%) infants had stage 1 ROP, 41 (61%) had was stage 2 ROP, and 14 (21%) had stage 3 ROP. ROP staging at discharge is detailed in Table 1. Eleven (16%) patients received intravitreal pharmacological treatment (9 ranibizumab, 1 bevacizumab, 1 afibercept).

**Table 1:** Stage of ROP at diagnosis and discharge in studied infants. Rate and (%)

	At diagnosis (n = 67)	At discharge^a^ (***n*** = 56)
**Stage 1**	12 (18)	7 (12)^b^
**Stage 2**	41 (61)	33 (59)^c^
**Stage 3**	14 (21)	16 (29)^d^
**Stage 4**	0	0
**Stage 5**	0	0

^a^ Six patients recovered from ROP; five patients were missed (four died and one was transferred to another hospital)

^b^ Five patients maintained the initial stage 1; two patients improved from stage 2

^c^ Twenty-nine patients maintained the initial stage 1; two patients worsened from stage 1; two patients improved from stage 3

^d^ Twelve patients maintained the initial stage 3; two patients worsened from stage 1; two patients worsened from stage 2

Infants with ROP had lower gestational age, birth weight and birth weight z-score, more frequent need of respiratory support, RBC transfusions, and sildenafil, more frequent complications, such as BPD, PDA, NEC, and PVL, and longer duration of stay in hospital (Table 2).

**Table 2:** Clinical characteristics of infants with and without ROP. Means + SD, or rate and (%), or median and (IQR)

	ROP (***n*** = 67)	No ROP (***n*** = 111)	P
**Gestational age (wks)**	26.6 ± 1.8	27.6 ± 1.5	< 0.001
**Birth weight (g)**	887 ± 268	1043 ± 262	< 0.001
**< 10° percentile**	9 (13)	7 (6)	< 0.001
**Z-score**	−0.05 ± 1.06	0.27 ± 0.87	0.874
**Male**	30 (44)	57 (51)	0.720
**Apgar score at 5th min**	8 (7–9)	8 (7–8)	0.609
**Antenatal steroids**	58 (87)	97 (87)	0.004
**Caesarean section**	48 (71)	81 (73)	0.310
**NIV**			
**Duration (h)**	27.5 (12.2–49.2)	16 (6–30)	0.002
**MV**			
**Duration (h)**	4 (0–21)	0 (0–2)	0.029
**RBC transfusions**	4.4 ± 5.5	1.6 ± 2.5	< 0.001
**Sildenafil**	10 (15)	4 (4)	
**Total dose (mg/kg)**	1.68 ± 2.59	0.12 ± 0.57	< 0.001
**Duration (d)**	20.1 ± 44.9	0.10 ± 0.51	< 0.001
**Maternal milk (%)**	40.3 ± 31.2	68.2 ± 31.1	< 0.001
**Human milk (%)**	9.2 ± 18.7	7.5 ± 13.2	0.477
**Formula milk (%)**	50.3 ± 29.6	24.1 ± 27.0	< 0.001
**Weight 36 weeks of PMA (g)**	1881 ± 434	1853 ± 414	0.691
**Z-score**	−1.78 ± 1.20	− 1.91 ± 0.94	0.434
**PDA**	39 (58)	62 (56)	0.035
**Sepsis**	26 (39)	35 (31)	0.053
**BPD**	24 (36)	25 (22)	0.025
**NEC**	7 (10%)	3 (3%)	0.048
**IVH,**			
**1–2 grade**	10 (15%)	13 (12%)	0.141
**3–4 grade**	11 (16%)	9 (8%)	
**PVL**	13 (19%)	10 (9%)	0.011
**Hospital stay (d)**	96.8 ± 55.4	65.0 ± 30.2	< 0.001
**Mortality**	4 (6%)	15 (13%)	0.156

*NIV* noninvasive ventilation, *MV* mechanical ventilation, *RBC* red blood cells, *PMA* post-menstrual age, *PDA* patent ductus arteriosus, *BPD* bronchopulmonary dysplasia, *NEC* necrotizing enterocolitis, *IVH* intraventricular hemorrhage, *PVL* periventricular hemorrhage

Regression analysis demonstrated that maternal milk (OR 0.979, 95% Cl 0.961–0.998) decreased the risk of developing ROP, while IVH (OR 2.055, 95% Cl 1.120–3.772) increased it, after adjusting for gestational age, birth weight (z-score), BPD, PDA, sepsis, NEC, PVL, RBC transfusions, and treatment with sildenafil. Moreover, we found that maternal milk (OR 0.981, 95% Cl 0.964–0.997) decreased the risk of ROP at discharge, while RBC transfusion (OR 1.522, 95% Cl 1.208–1.916) increased it.

## Discussion

In this study we evaluated the risk factors for ROP in a cohort of very preterm infants. We found an occurrence of any stage ROP of 38% which is similar to what has previously been reported [7–14]. In fact, epidemiological and cohort studies reported a ROP rate ranging from 23.2 [10] to 48.5% [12], with differences mainly due to different criteria for selecting studied populations.

We found that in our population the percentage of maternal human milk fed during infants’ hospital stay was correlated to the risk of developing ROP (both at the time of screening and at discharge) and had a protective effect. This result is in agreement with previous studies demonstrating that feeding with mother’s own milk can decrease the risk of ROP and severe ROP in very preterm infants [23, 24]. In a recent meta-analysis of five studies [only randomized controlled studies (RCTs)] involving 2208 preterm infants, Zhu et al. demonstrated that exclusive human milk versus exclusive formula feeding decreases the risk of any stage (OR 0.25, 95% Cl 0.13–0.49) and severe ROP (OR 0.10, 95% Cl 0.04–0.29), while mainly human milk versus mainly formula feeding decreases the risk of severe ROP (OR 0.16, 95% Cl 0.06–0.43) [23]. Moreover, Miller et al., in a meta-analysis of 40 studies (6 RCTs and 34 observational studies) involving 8778 preterm infants, demonstrated that higher vs. lower dose human maternal milk intake decreases the risk of severe ROP (Risk ratio 0.82, 95% Cl 0.70–0.98) [24].

Physiological mechanisms through which human milk may prevent the development of ROP may be the antioxidant [25] and immune-protective [26] properties of breastmilk. Human milk contains many antioxidant constituents, such as inositol, vitamin C, vitamin E, β-carotene, and essential fatty acids [i.e. docosahexaenoic acid (DHA)] [27, 28]. Among immunomodulatory substances, human milk contains secretory immunoglobulin A, lactoferrin, lysozyme, cytokines, oligosaccharides, antioxidant enzymes, and cellular components [28–30]. Moreover, human milk encompasses higher concentrations of insulin-like growth factor 1 (IGF-1) than preterm formulas [31], and it has been demonstrated that preterm infants fed with human milk have higher serum levels of IGF-1 [32]. This is particularly important since it has been shown that IGF-1 is crucial for physiological retinal vascularization, and its lack can lead to impaired retinal vascularity and ROP [4]. On the other hand, our patients received fresh, pasteurized, and/or frozen/thawed maternal milk and we are aware that these procedures may have changed its composition and affected the magnitude of its protective effect.

We found that the occurrence of any grade IVH increased the risk of ROP. This correlation was reported also by Yau et al. [10], while Chung et al. [12] found that IVH was correlated with the progression of ROP. This correlation can be explained by similar aspects of ROP and IVH pathogenesis, such as the vascular immaturity of retina and germinal matrix [33], and the role of oxidative stress induced by blood flow and oxygen delivery fluctuations which could promote hypoxic-ischemic and re-perfusion injuries and the development of these complications [34]. We did not find a correlation between IVH occurrence and the ROP at discharge. Therefore, our results suggest that IVH can favor the development of ROP but cannot affect its progression to worsening or improvement. Unfortunately, our data cannot explain why IVH can promote the development of ROP but not its outcome at discharge.

RBC transfusions was found to be a risk factors for ROP at discharge but not at the screening visit. This is consistent with the fact that the majority of transfusions for anemia of prematurity are performed after first weeks of life and, therefore, it can be speculated that they affect the progression of ROP rather than its onset. This correlation between RBC transfusions and ROP confirm previous findings [35, 36] which have been explained by the pro-oxidant effect of transfusions due to the increase in oxygen delivery to the retina secondary to increased packed cell volume and lower oxygen affinity of adult haemoglobin in packed red cells (PRCs), and secondary iron overload [37].

In our study, we could not demonstrate a role of gestational age and/or birth weight as risk factors for ROP as shown in previous studies [7–14]. This might depend on several factors, such as differences in studied populations, different model used for multivariable data analysis (i.e.: our model included the amount of human milk feeding and treatment with sildenafil which were not included in previous studies [7–14]), and a lack of severe ROP (>3rd stage) in our population. Thus, it is possible that the protective effect of human milk counteracted the detrimental effect of low gestational age and/or birth weight on ROP risk.

Limitations of our study include small sample size, variable medical and nursing care over time and between care teams and units, its retrospective design which precludes the possibility of assessing the role of other previously reported risk factors for ROP [38]. In particular, we did not evaluate some maternal factors because their role is controversial (i.e.: hypertensive disorders of pregnancy, premature rupture of membranes, chorioamnionitis, maternal diabetes, medications, age [38]) or because some data were not available (i.e.: maternal smoking, iron deficiency, anemia, blood leukocyte count [35]). Moreover, we did not evaluate some neonatal factors [38] because of their low (i.e: inhaled nitric oxide, erythropoietin, and dopamine treatment, hyperglycemia) or high (i.e.: caffeine treatment) occurrence in our population (unreported data) which did not allow comparisons between groups and evaluation of their possible effect. Another limitation of our study was the lack of severe ROP cases. This last point might decrease the comparability of our results with previous studies [7–14], but, on the other hand, this allowed us to highlight risk factors of mild-moderate cases of ROP which are commonly pooled with those of more severe cases assuming perhaps wrongly that they are the same.

## Conclusions

In conclusion, we found that the occurrence of ROP in our cohort was similar to what has previously been reported, and that feeding very preterm infants with higher volumes of maternal milk decreased the risk of its development while IVH occurrence increased it. Moreover, we found that maternal milk decreased the risk of ROP at discharge, while RBC transfusions increased it. These results suggest that effective strategies for promoting the use of mother’s own milk [39], preventing IVH [40], and standardizing the approach to RBC transfusions [18] can contribute to decreasing the risk of ROP in very preterm infants.

## Data Availability

The datasets used and/or analysed during the current study are available from the corresponding author on reasonable request.

## Abbreviations

BiPAP: bi-level NCPAP
BPD: bronchopulmonary dysplasia
HFNC: high-flow nasal cannulae
IVH: intraventricular hemorrhage
HFV: high frequency ventilation
NCPAP: nasal continuous airway pressure
N-IMV: nasal intermittent mechanical ventilation
NEC: necrotizing enterocolitis
NICUs: neonatal intensive care units
RBC: number of red blood cell
PVL: periventricular leukomalacia
ROP: retinopathy of prematurity
VIF: variance inflation factors
VEGF: vascular endothelial growth factor
